# Palmitic Acid Analogs Exhibit Nanomolar Binding Affinity for the HIV-1 CD4 Receptor and Nanomolar Inhibition of gp120-to-CD4 Fusion

**DOI:** 10.1371/journal.pone.0012168

**Published:** 2010-08-13

**Authors:** Elena E. Paskaleva, Jing Xue, David Y-W. Lee, Alexander Shekhtman, Mario Canki

**Affiliations:** 1 Center for Immunology and Microbial Disease, Albany Medical College, Albany, New York, United States of America; 2 Department of Chemistry, State University of New York at Albany, Albany, New York, United States of America; 3 Mailman Research Center, McLean Hospital, Harvard Medical School, Belmont, Massachusetts, United States of America; Tsinghua University, China

## Abstract

**Background:**

We recently reported that palmitic acid (PA) is a novel and efficient CD4 fusion inhibitor to HIV-1 entry and infection. In the present report, based on *in silico* modeling of the novel CD4 pocket that binds PA, we describe discovery of highly potent PA analogs with increased CD4 receptor binding affinities (K_d_) and gp120-to-CD4 inhibition constants (K_i_). The PA analogs were selected to satisfy Lipinski's rule of drug-likeness, increased solubility, and to avoid potential cytotoxicity.

**Principal Findings:**

PA analog 2-bromopalmitate (2-BP) was most efficacious with K_d_ ∼74 nM and K_i_ ∼122 nM, ascorbyl palmitate (6-AP) exhibited slightly higher K_d_ ∼140 nM and K_i_ ∼354 nM, and sucrose palmitate (SP) was least efficacious binding to CD4 with K_d_ ∼364 nM and inhibiting gp120-to-CD4 binding with K_i_ ∼1486 nM. Importantly, PA and its analogs specifically bound to the CD4 receptor with the one to one stoichiometry.

**Significance:**

Considering observed differences between K_i_ and K_d_ values indicates clear and rational direction for improving inhibition efficacy to HIV-1 entry and infection. Taken together this report introduces a novel class of natural small molecules fusion inhibitors with nanomolar efficacy of CD4 receptor binding and inhibition of HIV-1 entry.

## Introduction

Recently we isolated and identified palmitic acid (PA) as a novel natural small molecule that inhibits HIV-1 fusion and infection by the mechanism of binding to the CD4 receptor and blocking gp120-to-CD4 attachment [1], [2], [3]. We showed that PA binds to the CD4 receptor with K_d_ ∼1.5 µM [1], and it blocks gp120-to-CD4 attachment with K_i_ ∼2.53 µM (submitted for publication). PA also inhibited R5-tropic HIV-1 infection in cervical explant model of human vagina and in the underlying cervical submucosa primary PBL and macrophage cells, without toxicity (submitted for publication). Collectively, these results indicated potential for PA's microbicide development.

However, the efficacy of HIV-1 inhibition by PA remains in submicromolar range, suggesting potential for improving its efficacy. The PA molecule binds to the CD4 receptor *via* its hydrophobic methyl and methelene groups located away from the PA carboxyl end, and the carboxyl group functions by blocking efficient gp120-to-CD4 attachment and fusion [1]. Considering PA's molecular structure and bifunctional mechanism of inhibition, we *in silico* modeled this structure-activity relationship (SAR), and we searched chemical databases for PA analogs that would satisfy Lipinski's rule of drug-likeness [4]. In the present study we report nanomolar CD4 binding affinities and nanomolar blocking efficacies of gp120-to-CD4 fusion, by three analogs of PA: 2-BP, 6-AP, and SP.

## Results

### PA and gp120 binding sites on CD4 overlap

**Figure 1:**
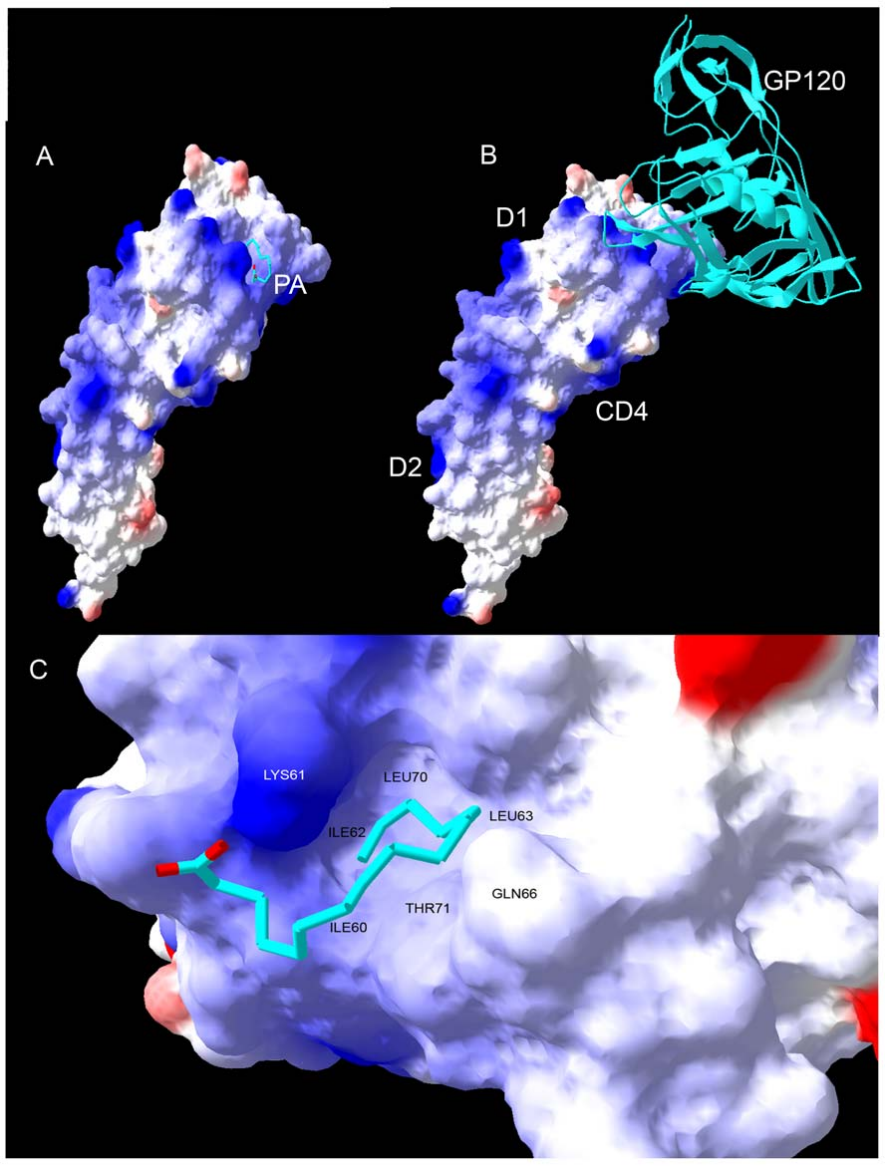
PA-CD4-gp120 interaction model. **A**) Molecular docking software Autodock 4.0 was used for blind docking of flexible PA onto rigid two N-terminal domains of CD4 (PDB code 1GC1). The resultant PA-CD4 conformations were ranked and categorized based on the value of free energy of binding. 386 out of 1000 docking runs fell into conformations that are ranked with the highest score (−16 kcal/mol). The root mean square deviation of these conformations was 1.2 A suggesting very similar binding modes. One of the ligand bound conformations of PA-CD4 with a highest score (−17 kcal/mol) is shown in cyan (PA aliphatic chain) and red (PA carboxylic terminus). **B**) Crystal structure of gp120-CD4 (PDB code 1GC1). The backbone of gp120 is shown by using ribbon model. The N-terminal D1 and D2 domains of CD4 are indicated. Comparison between PA-CD4 and gp120-CD4 structures shows the overlapping binding sites for gp120 and PA. **C**) Close-up of the PA-CD4 binding cavity shown in A. PA occupies this cavity, which is formed by Phe52, Ile60, Ile62, Leu63, and Leu70 of CD4. Electrostatic potential calculated using DelPhi software (B. Honnig's Lab) was mapped onto the molecular surface of CD4. Positively and negatively charged surfaces are in blue and red, respectively. Non-polar surface is in white. We used Discover Studio software (AccelRys) to prepare this figure.

To gain structural insights into the mechanisms of PA binding to the CD4 receptor, we used *in silico* molecular docking based on the known X-ray structure of the two N-terminal domains of CD4 (aa 26-206) (PDB code 1GC1) and a flexible PA ligand (Figure 1). We used Autodock 4.0 molecular docking program [5], [6], which is widely used to identify a ligand binding contiguous envelope of maximum affinity for a given macromolecular structure. The geometry of PA-CD4 with a highest score is shown in Figure 1A, and crystal structure of gp120-CD4 (PDB code 1GC1) binding is shown in Figure 1B. Comparison between PA-CD4 and gp120-CD4 structures shows the overlapping binding sites for gp120 and PA, suggesting that PA directly inhibits complex formation between CD4 and gp120 that is necessary for HIV-1 entry. To access the importance of hydrophilic and hydrophobic interactions between PA and CD4, in a close-up of the PA-CD4 binding cavity we mapped CD4 electrostatic potential onto the molecular surface, with blue and red colors representing positively and negatively charged surfaces, respectively (Figure 1C). PA hydrophobic aliphatic chain fits tightly into the CD4 binding cavity, formed by Phe52, Ile60, Ile62, Leu63, and Leu70. The negatively charged carboxylic group of PA is in the vicinity of the positively charged epsilon amino group of Lys61. PA methelene groups proximal to the PA carboxyl end do not make extensive contacts with the CD4 binding cavity and are possibly flexible. These results are in agreement with our previous STD NMR results, which identified PA binding epitope on CD4 that consists of the hydrophobic aliphatic chain located away from the PA carboxyl end [1].

### PA analogs bind to CD4 receptor with nanomolar affinity

We hypothesized that based on the CD4-PA model, by adding polar groups to the carboxyl terminal of PA as well as by creating partially negative charge on the hydrocarbon chain close to carboxyl end we would increase the affinity of PA for CD4. We used the following criteria in designing PA analogs: 1) The compounds should be non-toxic, 2) The compounds should be readily available from the chemical catalogs, and 3) The compounds should satisfy Lipinski's rule of five for solubility, lipophilicity, and hydrogen bond formation. We chose three compounds that meet these criteria: SP and 6-AP, and 2-BP. 6-AP is used as a substitute for vitamin C, is approved for human consumption, and is available over the counter (OTC) without prescription in most pharmacies and health food stores. SP is a non-toxic sugar ester of PA that is used in food and cosmetics industry, and although it does not satisfy the Lapinskis's rule, we used it because of its increased solubility. 2-BP is a non-toxic compound used to inhibit palmitoylation [7], [8]. As compared to PA, these compounds have favorable physico-chemical properties shown in Table 1 and Table S1. Higher solubility, critical micelle concentration (cmc), and lower lipophilicity of these PA analogs than PA are important to increase efficacy of the compounds against HIV-1 in human tissue and minimize possible cytotoxic effects.

**Table 1:** Physico-chemical properties of PA analogs.

Compound	Chemical name	Solubility*,µM	CLogP**	cmc***,µM
PA	Palmitic acid	28	7.2	4
2-BP	2-Bromohexadecanoic acid	40	3.46	5
6-AP	6-O-Palmitoyl-L-ascorbic acid	32	5.8	7
SP	Sucrose palmitate	100	4.56	30

*Solubility was calculated directly from chemical structure by using ACD/PhysChem program (ACD/Lab, Inc) [13].

**Computed partition coefficients (CLogP) were calculated directly from chemical structure by using ACD/PhysChem program (ACD/Lab, Inc) [13].

***Critical micelle concentration (cmc) was determined experimentally by analyzing changes in 1D proton NMR spectra of the compounds due to aggregation [14].

**Figure 2:**
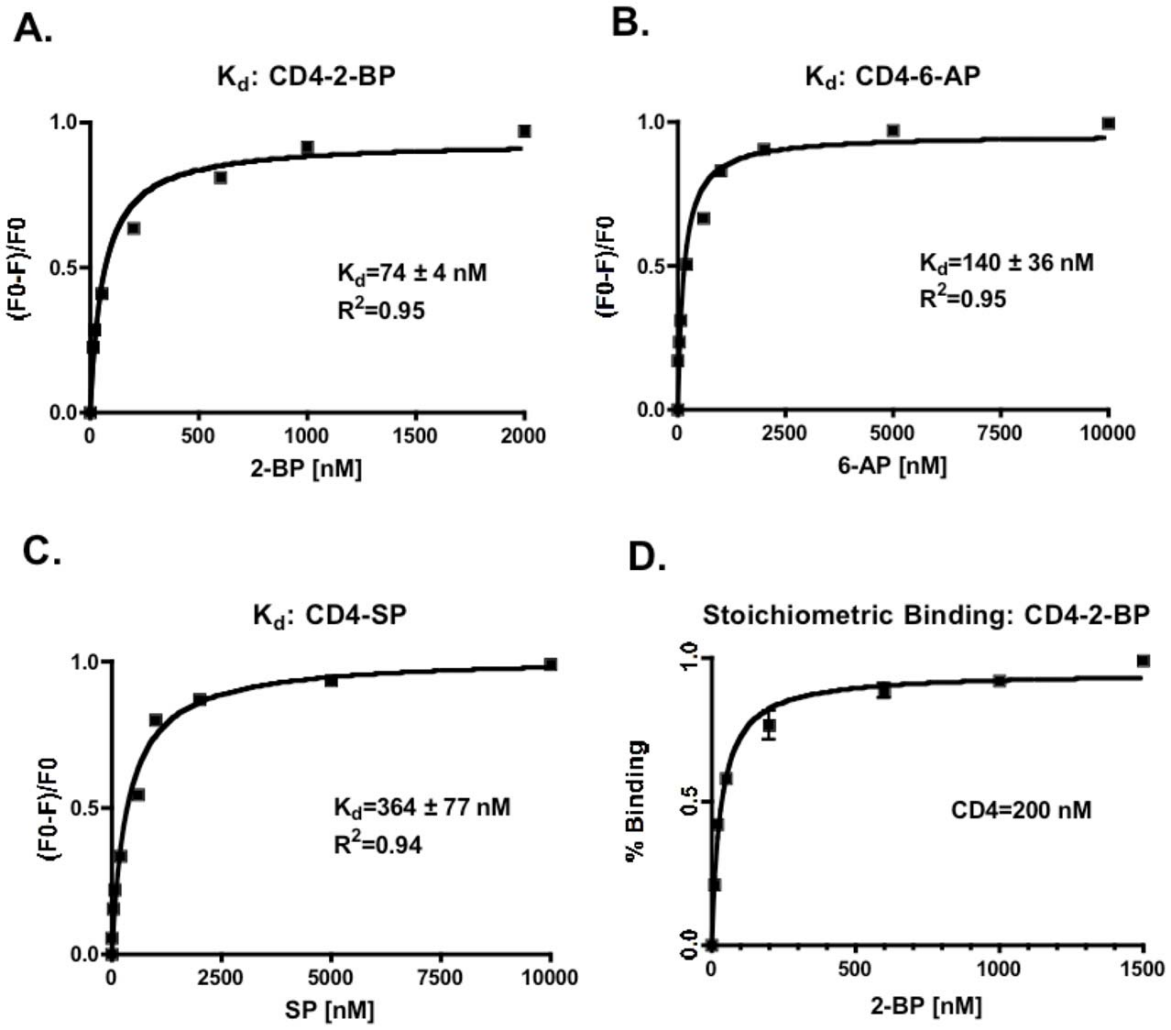
*In vitro* experiments of PA analogs binding to sCD4. Binding isotherm of the sCD4 tryptophan fluorescence at the wavelength of 350 nm with the increasing concentration of **A**) 2-BP, **B**) 6-AP, and **C**) SP; **D**) stoichiometric binding of 2-BP to sCD4. Tryptophan fluorescence was measured using an excitation wavelength of 280 nm. In all cases, we observed tryptophan fluorescence quenching of sCD4 by the increasing concentration of PA analogs. During stoichiometric binding experiment the concentration of sCD4 was kept at 200 nM, which is well above the dissociation constant of 2-BP-sCD4, 74 nM. Curve fitting (OriginLab) was performed to find the best values for *K_d_* using a single site binding isotherm approximation.

The tryptophan fluorescence of the soluble extracellular portion of CD4 (sCD4) was used to estimate binding affinity of PA analogs for sCD4 *in-vitro* (Figure 2). Saturating concentrations of PA analogs, 2-BP, 6-AP, and SP quenched 50%, 40%, and 60% of the sCD4 tryptophan fluorescence, respectively, and resulted in a red shift of the emission peak of 4, 3, and 5 nm, respectively. Based on the fluorescence titration experiments, dissociation constant, K_d_, for 2-BP, 6-AP and SP was estimated to be ∼74±4 nM, 140±36 nM, and 364±77 nM (Figure 2 A, B, and C, respectively).

Since PA analogs form micelles at micromolar concentration it was important to characterize binding stoichiomentry of PA analogs to CD4 (Figure 2D). We estimated binding stoichiometry of the most potent PA analog, 2-BP, by using the fluorescent titration experiment. The concentration of sCD4 was kept at 200 nM, which is well above the 74 nM dissociation constant of 2-BP-CD4. In this case, titrated 2-BP was fully bound. The observed changes in sCD4 fluorescence would level off when 2-BP-sCD4 reached proper stoichiometry. Binding of 2-BP to sCD4 resulted in fluorescence quenching that was saturated when the concentration of 2-BP reached 200 µM, indicating that stoichiometry of 2-BP-CD4 is 1∶1. This result proved that 2-BP does not form micelles when it binds directly to CD4.

### PA analogs inhibit *in vitro* gp120-to-CD4 complex formation

**Figure 3:**
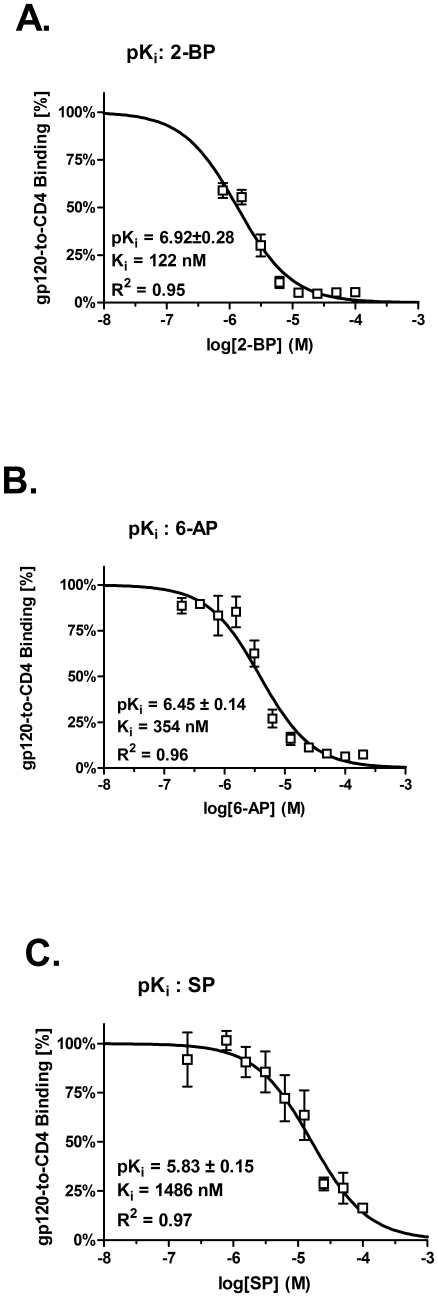
Inhibition of gp120-CD4 complex formation. Inhibition of gp120-CD4 complex formation was investigated by gp120 capture ELISA. Envelope gp120 (IIIB) protein was captured on 96 well plates, washed, and incubated in the presence of CD4-biotin alone or in the presence of increasing concentrations of A) 2-BP, B) 6-AP, or C) SP, as indicated. Strepavidin-HRP was added, and then developed by addition of o-Phenylenediamine dihydrochloride (OPD) substrate. Colorimetric reaction was stopped by adding 1N HCl, and read at 490 nm. Percent of gp120-CD4 binding was calculated from gp120-CD4 complex formation in the absence of any inhibitor. pK_i_ (-log K_i_, M) was calculated and plotted in Prizm (GraphPad Software), and inhibition constant, K_i_, was calculated by using the equation K_i_ = IC_50_/(1+[CD4]/K_d_) [15], based on IC_50_ concentration of bound CD4, [CD4]  = 50 nM, and CD4 binding affinity for gp120, K_d_ = 5 nM. Representative of three experiments, all data are mean ± SD.

To determine PA analogs inhibition constant for blocking gp120-to-CD4 attachment that is independent of *in vivo* cellular CD4 expression, we performed *in vitro* gp120 capture ELISA (Figure 3 A–C). 96-well plates were coated with gp120 (IIIB) and incubated with biotin-sCD4 in absence or presence of increasing PA analogs concentrations, as indicated. All three PA analogs inhibited CD4 attachment to HIV-1 X4-tropic (IIIB) gp120 envelope in a dose dependant manner. 2-BP, 6-PA, and SP blocked gp120-CD4 complex formation with Ki of ∼122, 354, and 1486 nM (Figure 3 A, B, and C, respectively). These results are consistent and validate our CD4 binding affinities results (Figure 2). In reverse experiments, we coated plates with sCD4, incubated gp120 with increasing concentrations of 2-BP, 6-AP, and SP, and tested for inhibition of gp120-CD4 complex formation, which was not inhibited, indicating that PA analogs do not bind to soluble gp120 (not shown). This result is also consistent with previously published observation that PA does not bind to gp120 [1].

### PA analogs are not toxic to *in vitro* cell culture

**Figure 4:**
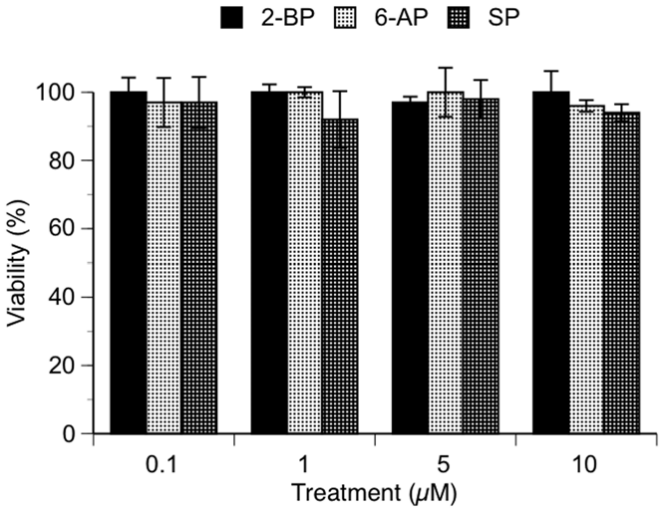
Viability of PA analogs in tissue culture. 1G5 cells were treated with increasing concentrations of 2-BP, 6-AP, and SP, as indicated, and cell viability was determined 72 h. after treatment by MTT assay. % Viable cells were calculated from untreated mock control, which was taken as 100% viable. Bars indicate ± SD, representative of 2 experiments.

To ascertain possible toxicity of PA analogs, we tested each analog in cell culture system (Figure 4). 1G5 cells were treated with increasing concentrations of 2-BP, 6-AP, or SP, from 100 to 10,000 nM, and viability was determined by MTT assay at 24 h. (not shown) and at 72 h (Figure 4). Viability for all treatments and at all concentrations tested, remained ≥92% for up to 72 h. of follow-up. These results are consistent with predicted safety for these PA analogs and lack of toxicity previously reported for PA in primary PBL and macrophages [1].

## Discussion

We have demonstrated that palmitic acid is a novel class of small molecule that binds to the CD4 receptor and blocks gp120-to-CD4 fusion and HIV-1 infection. We determined K_d_ for the CD4 receptor to be ∼1.5 µM [1]. In primary PBL and macrophages, PA blocked productive X4 and R5-tropic HIV-1 infection with IC_50_ of ∼0.34 and 33 µM, respectively [1]. We also showed that PA inhibited R5 HIV-1 infection in human *ex vivo* model of human vagina, demonstrating opportunity for a microbicide development (submitted for publication).

However, restricted sub-micromolar inhibition efficacy, limits PA's clinical utility. To this end we were interested to investigate the possibility of PA to serve as a model scaffold molecule for chemical modifications to improve its efficacy to nanomolar range. We constructed *in silico* model of PA-CD4 binding and gp120 interaction (Figure 1). This model was supported by our STD-NMR and gp120 capture ELISA results, which collectively showed that PA binds to the CD4 receptor *via* hydrophobic methyl and methelene groups located away from the PA carboxyl end, and the carboxyl group functions by blocking efficient gp120-to-CD4 attachment and fusion [1]. Tight fit of PA into the CD4 cavity formed by Phe52, Ile60, Ile62, Leu63, and Leu70 possibly precludes extensive modifications of the PA aliphatic chain. At the same time, modifying the PA carboxyl end and methylene groups close to the carboxyl end may lead to increase in the PA-CD4 affinity, and adding bulky groups to the carboxyl end may also increase the ability of PA to block CD4-gp120 interaction (Figure 1). Based on this model and utilizing Lipinski's rule of drug-likeness we searched available chemical databases and investigated three different PA analogs (Table 1), for their CD4 binding affinities and gp120-to-CD4 inhibition constants that are independent of *in vivo* CD4 receptor expression. All three compound showed nanomolar CD4 binding affinity and nanomolar gp120-CD4 blocking efficacy (Figure 2 and 3), and were not toxic to *in vitro* cell culture, up to 10,000 nM concentrations for 72 hours (Figure 4). Because PA analogs form micelles it was important to characterize binding stoichiomentry of PA analogs to CD4 (Figure 2D). Our results showed that the most efficacious 2-BP analog, bound to CD4 with a 1∶1 binding stoichiomentry. This result signifies that PA analogs inhibit HIV-1 fusion by binding to the CD4 receptor as a single molecule, and not in the form of micelles that exist at higher micromolar concentrations. Collectively, our results demonstrate that PA may serve as a model scaffold molecule to further modify its structure and improve its efficacy. Considering we only tested commercially available analogs, we predict that further chemical modifications based on PA's known SAR, will lead to additional increase in inhibition efficacy. However, considering successes of HAART treatment in AIDS patients, it is not unreasonable to envision utility of PA based fusion inhibitor to be combined together with antiretroviral drug targeting a different stage of the virus life cycle, which may result in an effective and potent inhibitor of HIV-1 infection.

## Materials and Methods

### Reagents

All reagents and solvents were obtained from commercial suppliers and used without further purification. sCD4 (Progenics Pharmaceuticals, Inc) was 95% pure, according to manufacture's specification. The specified chemical purity of 2-BP (2-Bromohexadecanoicacid, Sigma-Aldrich), 6-AP (6-O-Palmitoyl-L- ascorbic acid, Sigma-Aldrich) and SP (sucrose palmitate, Stearinerie Dubois, France) were better than 98%. 1G5 [9] cells were obtained from HIV AIDS Research and Reference Reagent Program, Division of AIDS, NIAID, NIH, and were cultured and maintained as specified by the reagent protocol.

### *In silico* modeling

We used a standard protocol for Autodock–based blind docking approach described and successfully implemented by Hetenyi et al [10]. AutoDock 4.0 uses a force-field based empirical free energy scoring function [5]. The Lamarckian Genetic Algorithm (LGA) [11] was used as a search engine. The active site was defined using AutoGrid. The grid size was set to 30 Å×30 Å×30 Å points with grid spacing of 0.3 Å centered on the CD4 D1 domain center of mass. Step sizes of 1.0 Å for translation and 50 degree for rotation were chosen, a maximum number of energy evaluations was set to 4 million. PA was prepared as a flexible ligand possessing 14 torsional rotations and CD4 was treated as a rigid target. The resultant PA-CD4 conformations were ranked and categorized based on the value of free energy of binding. The AutoDock free energy of binding ranged from −17 kcal/mol to −5 kcal/mol. 386 out of 1000 docking runs fell into conformations that are ranked with the highest score (−16 kcal/mol). The root mean square deviation of these conformations was 1.2 Å suggesting very similar binding mode. We used Discover Studio software (AccelRys) to prepare the figure.

### Fluorescence Titration

Measurements were performed on a Fluorolog-3 fluorescence spectrophotometer (HORIBA Jobin Yvon) at 25°C in a 1-ml stirred cuvette. For fluorescence titration experiments, 100 nM of sCD4 dissolved in 10 mM phosphate buffer [pH 7.4] and 250 mM NaCl was used, and 100 µM solution of PA analogs, 2-BP, 6-AP, and SP dissolved in dimethylsulfoxide (DMSO) was added in 10 nM steps. Titrations in the absence of sCD4 and in the absence PA analogs were performed as reference. Tryptophan fluorescence was measured using an excitation wavelength of 280 nm. The fluorescence emission signal was subtracted from the signal of the reference titrations, and the differences adjusted by the dilution factor were plotted against the final concentration of added PA analogs. Curve fitting (OriginLab) was performed to find the best values for *K_d_* using a single site binding isotherm approximation [12].

### gp120-CD4 capture ELISA

Inhibition of gp120-CD4 complex formation was investigated by CD4-to-gp120 capture ELISA, in accordance with manufacturer's instructions (ImmunoDiagnostics, Inc., MA), and as previously described [1]. Briefly, envelope gp120 (IIIB) protein (ImmunoDiagnostics, Inc., MA) was captured on 96-well plates, washed, and incubated in the presence of 50 nM biotin-conjugated sCD4 (Immunodiagnostics) alone or in the presence of serial dilutions of PA analogs, as indicated. Strepavidin-HRP was added, and then developed by addition of OPD substrate. Colorimetric reaction was stopped by adding 1N HCl, and read at 490 nm. Percent of gp120-CD4 binding was calculated from gp120-CD4 complex formation in the absence of any inhibitor.

### *In vitro* cell culture toxicity of PA analogs

For determination of viability, 1G5 cells were seeded in 12 well plates at 1×10^6^ cells/well, treated with increasing concentrations of PA analogs, from 100 to 10,000 nM (0.1 to 10 µM), as indicated. After 24 and 72 hours, cells were collected and analyzed for viability by a CellTiter 96 Non-Radioactive Cell Proliferation Assay [(3-(4,5-Dimethyl-2-thiazolyl)-2,5 dephenyltetrazolium, Promega,] (MTT) assay, as specified by the manufacturer, and as previously described [2].

## Supporting Information

Table S1 (1.10 MB TIF)
