# Supplementary figures and images for: Palmitic Acid Analogs Exhibit Nanomolar Binding Affinity for the HIV-1 CD4 Receptor and Nanomolar Inhibition of gp120-to-CD4 Fusion
Source: PLoS One. 2010 Aug 13;5(8):e12168. doi: 10.1371/journal.pone.0012168 (PMC2921400; doi:10.1371/journal.pone.0012168)

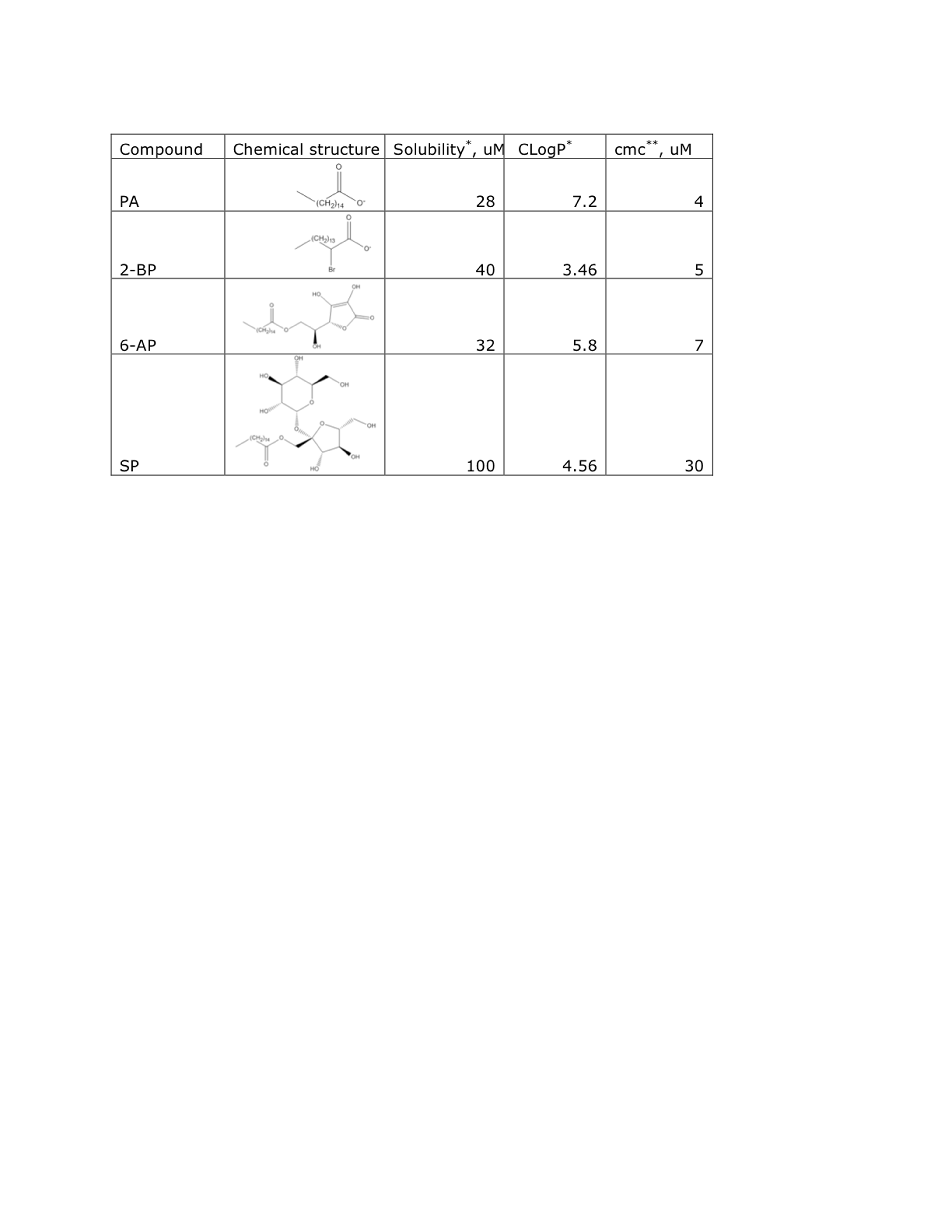

Supplement: Table S1 — (1.10 MB TIF) [file pone.0012168.s001.tif]
